# It's a Match, Isn't It? Developing a Checklist on Matching Secondary Data at the Example of Job Control

**DOI:** 10.1002/ajim.70135

**Published:** 2026-10-03

**Authors:** Franziska J. Kößler, Candice Y. Johnson, Amel Omari, Kaori Fujishiro

**Affiliations:** ^1^ Institute of Management & Psychology, School of Management and Technology, Leuphana Universität Lüneburg Lüneburg Germany; ^2^ Department of Epidemiology and Biostatistics Michigan State University East Lansing Michigan USA; ^3^ Independent Scholar Cincinnati Ohio USA; ^4^ Division of Field Studies and Engineering (retired) National Institute for Occupational Safety and Health Cincinnati Ohio USA

## Abstract

Few datasets simultaneously capture occupational exposures and health outcomes in a general working population. To understand associations between occupational exposures and health, researchers often combine existing datasets. As occupation codes are available in many datasets, combining these datasets seems a straight‐forward option. However, the different purposes of these datasets and correspondingly their coding systems may complicate and bias matching procedures by dropping occupations, squeezing multiple occupations into one category, and linking them incorrectly. To develop a checklist that supports an entire research and writing process based on matching datasets, we demonstrate problems that may occur when matching three datasets based on existing crosswalks. Two of these datasets provide unique occupational information: occupational exposure (specifically, job control) from the Occupational Information Network (O*NET) and sociodemographic composition of occupations from the American Community Survey (ACS). After matching them to each other, we used them to enrich workers' health data from the General Social Survey (GSS). This matching process showed that 993 detailed O*NET‐SOC codes from 2019 (O*NET‐19 codes) matched 420 U.S. Census occupation codes (Census codes). This reduction resulted from two situations: a lack of equivalent Census codes and a consolidation of several O*NET‐19 codes under a single Census code. Although we could match occupational data to all workers in GSS, full information on occupational exposure and sociodemographic composition of their jobs was not available for 35% of them. Since our findings are limited to O*NET‐based rating of job control, we encourage other researchers to explore scoring of other aspects of occupational exposure and provide a checklist for that purpose.

## 1 Introduction

Using existing datasets for research is cost and time‐efficient, but not all needed information is found in one place. This is especially true for health researchers who want to examine occupational exposure and health in a general working population. Occupational exposure data are often collected within a specific industry or occupation (e.g., radiation exposure among workers at nuclear power plants). Consequently, these samples do not represent a general working population, and the information collected often lacks detailed health or sociodemographic information. Population health surveillance datasets (e.g., Behavioral Risk Factor Surveillance System [1], and the National Health and Nutrition Examination Survey [2]), on the other hand, have rich information on individual characteristics and health but seldom include occupational exposure data. This misalignment in data availability hinders inquiries on occupational exposure in population health research [3, 4].

To investigate the distribution of occupational exposure and health across sociodemographic groups in the general population, we need data on occupational exposure, a health indicator of interest, sociodemographic characteristics, and other variables that allow us to explore alternative explanations (i.e., mediation), confounding, or effect modification. If multiple datasets, each containing some of the needed variables, all included occupation codes as a common key, then they could be merged for an analysis. However, this process of merging datasets can be complicated.

Complications arise when various datasets use different job coding systems. Although they all reference jobs people do, different systems such as the Standard Occupational Classification (SOC) [5, 6] codes and U.S. Census Occupation Codes [6] are not directly compatible because SOC is generally more detailed than Census codes.^1^ For example, cooks in a fast food restaurant (35‐2011), institution and cafeteria (35‐2012), private household (35‐2013), restaurant (35‐2014), short order (35‐2015), and all other (35‐2019) are assigned separate SOC codes but are all summarized under one Census code, 4020, for cooks [7, 8]. When we try to match occupational codes from different systems, some jobs need to be combined, and others may be dropped. The Census Bureau provides crosswalks between SOC and Census codes as well as those from historical revisions [9]. These crosswalks are useful in creating a common key across multiple datasets so that they can be used for population health research. However, we must be aware of how exactly codes are matched because logistical details—combining and dropping jobs—result in missing data and may create a biased dataset for the analysis.

Complications may also arise when different versions of a system need to be merged. As the economy shifts, different versions reflect emerging and disappearing jobs (e.g., “precision typesetters” was removed from SOC in 2000 [10], while “life scientists, all other” appeared in Census in 2010 [11]); as well, older jobs are summarized by a single new job code (e.g., “farmers, ranchers, and other agricultural managers” is based on two Census codes from 2000 [11]) or split into multiple novel ones (e.g., “computer scientists and systems analysts” was split into four different codes in 2010 [11, 12, 13]). Thus, matching different versions of the same coding system may involve similar logistic problems as matching across coding systems, despite considerable efforts of the responsible authorities in harmonizing them (for details, see Scopp [12]).

There are at least two ways in which potential biases could be created in a combined dataset: one related to the sociodemographic representativeness of the final sample, and another related to the range and level of the exposure of interest. In the process of matching datasets, if jobs that are mostly held by a sociodemographically defined group (e.g., women, people with high school diploma) cannot be matched and therefore must be dropped, then the final dataset underrepresents the group. Similarly, if jobs with a higher or lower exposure level of the occupational hazard of interest were unmatched and thus dropped, the final dataset would produce incomplete results. Moreover, if multiple jobs with different levels of exposure have to be combined, then the averaged exposure measure introduces exposure misclassification, potentially over‐ or underestimating the level of exposure for some jobs (i.e., information bias).

### 1.1 Context and Overview of This Commentary

In this commentary, we show step by step how individual‐level health data can be enriched by information on occupational exposure and workforce sociodemographics. We match three datasets and evaluate the result and its implications at each step. Based on specific problems we encountered in this process, we clarify how potential problems arise and suggest responsible ways to make use of existing information by providing a checklist for researchers. For occupational exposure data, we used the Occupational Information Network (O*NET) database [14]. To obtain the sociodemographic composition and thus representativeness of each job, we used the American Community Survey (ACS) [15], accessed through the Integrated Public Use Microdata Series (IPUMS) [16]. For health and other individual characteristics, we used the General Social Survey (GSS) [17].

As shown in Figure 1, this combination involved three steps: In the first step, we cross‐walked the 2019 O*NET‐SOC codes (via 2010 O*NET‐SOC codes and 2010 SOC codes)^2^ to Census codes in order to be able to match O*NET with the other datasets (i.e., we simplified the coding). In the second step, we matched occupational exposure data from O*NET with occupational sociodemographic data from ACS to create *occupation data* by Census codes (i.e., the unit of analysis here is the occupation, and variables describe the characteristics of occupations). In the third step, we matched this occupation dataset (i.e., the combination of O*NET and ACS) to worker health data (from GSS). In the resulting *enriched worker health data*, the unit of analysis is the individual, and variables describe individual workers' health as well as the characteristics of the job each individual has. Yet, individual workers within an occupation share the same level of occupational exposure related to clustering by occupations [14].

**Figure 1:**
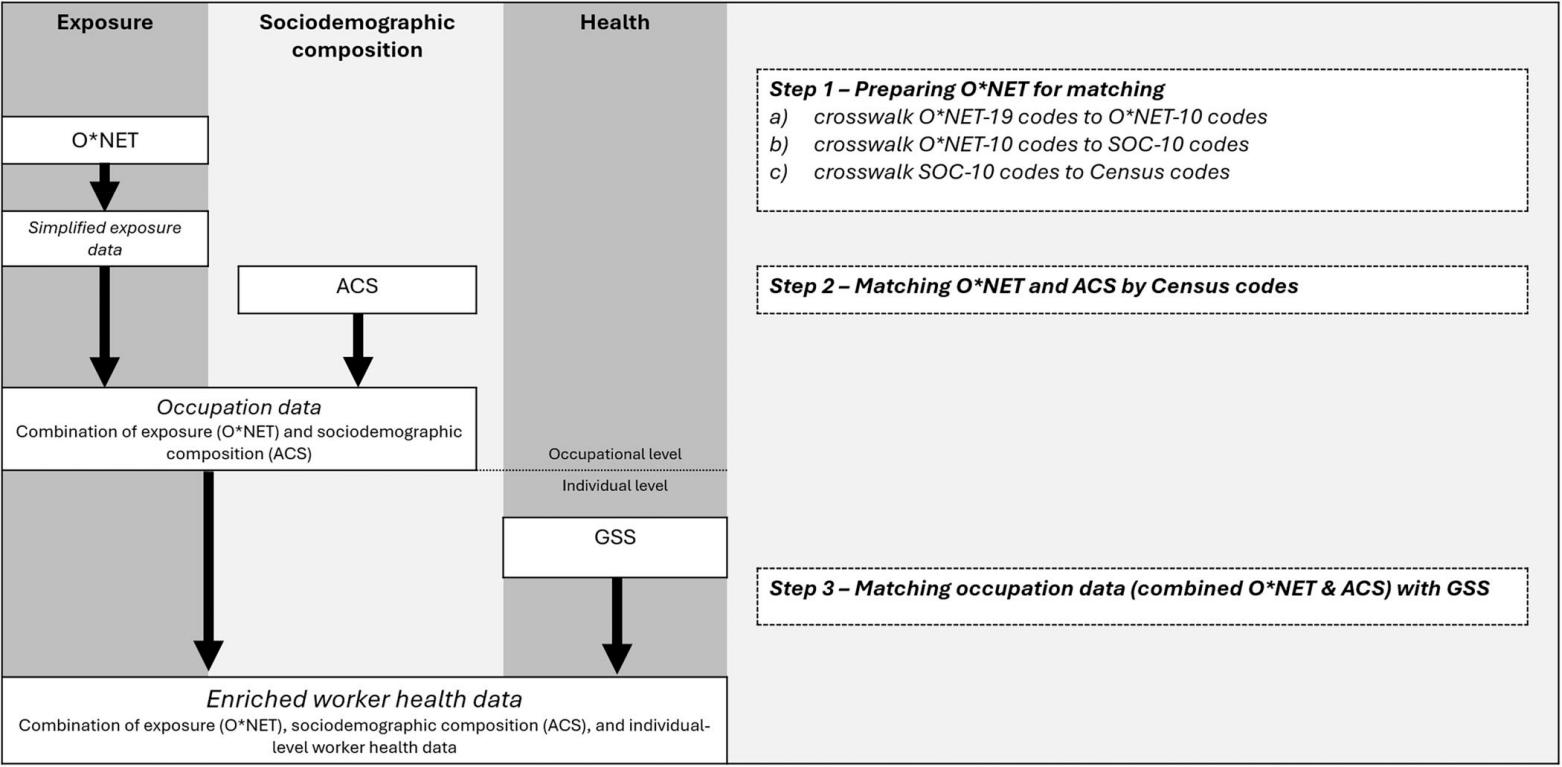
Visualization of the matching process. *Note*: O*NET = Occupational Information Network. O*NET‐10 codes = 2010 Standard Occupational Classification codes with O*NET‐extension. O*NET‐19 codes = 2019 Standard Occupational Classification codes with O*NET‐extension. SOC‐10 codes = 2010 Standard Occupational Classification codes. Census codes = Census 2010 occupation codes. ACS, American Community Survey; GSS, General Social Survey.

Applying this three‐step crosswalk across three datasets, we explore how jobs that can be matched differ from jobs that cannot be matched in terms of job control as exposure (O*NET) and sociodemographic composition (ACS). We chose job control as the exposure because it is a commonly studied psychosocial characteristic that is relevant to workers’ health [18, 19]. Additionally, we decided to explore occupational sociodemographics (e.g., sex and race composition) to understand whether jobs primarily held by specific groups are systematically excluded. Researchers may decide for themselves which steps of the process outlined here are useful for them based on their research question and the information included in the datasets; at some places, we also point out which steps can be skipped for a specific interest. Based on our evaluation of this standard matching process, we developed a checklist researchers and reviewers might use to deal with problems that occur in this process.

## 2 Methods

### 2.1 Datasets and Measures

#### 2.1.1 Job Coding System: Census 2010 Occupation Codes

Since we aimed to enrich individual workers’ health data with occupational data, the job coding system used in the individual workers' health data (Census codes) was the main linkage among our datasets. Because Census codes are not the most detailed, linking data using Census codes involves a compromise (i.e., loss of details) and potentially introduces exposure misclassification and information bias.

#### 2.1.2 Occupational Exposure Data: O*NET

Developed by the U.S. Department of Labor as a tool for helping job seekers to find jobs that match their skills and interests, the O*NET database provides detailed job task characteristics of over 900 jobs in the US economy. To update this information annually, data have been collected continuously since 1998 from various sources. Experts, named by professional and educational organizations, rate the abilities a job requires. Additionally, randomly selected workers from randomly selected workplaces complete questionnaires on their current job tasks, activities, and working environments. These data are then aggregated at the occupation level (see also the O*NET Resource Center [20] and RTI International, responsible for data collection [21]). Thus, O*NET allows researchers to compare occupations on various topics, such as job control, income levels, job prospects, and required skills [22]. For this demonstration, we used O*NET version 29.0 published in August 2024 [23]. This version provides information on 993 jobs based on O*NET‐19 codes; these are SOC‐18 codes that have an O*NET‐specific two‐digit extension.

We focused on the O*NET variables that reflect job control, a combination of decision authority and skill discretion [18]. More specifically, we operationalized O*NET job control using the variables *structured versus unstructured work* and *freedom to make decisions* from the work context module (4.C.3.b.8 and 4.C.3.a.4) as well as the variables *importance and level of updating and using relevant knowledge* from the work activity module (4.A.2.b.3 IM and LV) [23]. All items are measured on a scale from 1 to 5,^3^ except for the level of updating and using knowledge (4.A.2.b.3 LV), which is measured on a scale from *low* (0) to *high* (7) levels. Therefore, we harmonized them so that all items had a theoretical range from *low* (0) to *high* (100) [24]. Then we calculated the mean score for updating and using knowledge and included it in a mean score with the other two items. Job control estimates were available for 879 of 993 (88.5%) O*NET‐19 codes.

#### 2.1.3 Sociodemographics of Occupations: ACS

IPUMS USA provides access to ACS data, including sociodemographic and occupational data [16]. Since 2001, these data have been collected annually at the individual level. The latest ACS data is based on a stratified random sample of about 0.4%–1% of the US population.

We derived summary statistics from IPUMS based on the Survey Documentation and Analysis system [25].^4^ We selected the years 2002, 2006, 2010, 2014, 2018, and 2022 (i.e., the years when GSS assessed occupational and health data) as well as people who worked in the last year in civilian occupations.^5^ For this population, we obtained the distribution of age, income, sex, race/ethnicity, and education within each Census code (see Online Supplement B).

#### 2.1.4 Workers' Health Data: General Social Survey

Since 1972, GSS has been monitoring opinions, attitudes, behaviors, health status, and lifestyle factors biannually in a representative cross‐sectional sample of adults in the United States. As occupational health scientists may benefit the most from the Quality of WorkLife module (QWL) [26], we focused primarily on this module that assessed data on physical as well as psychosocial working conditions and occupational well‐being every four years since 2002 (i.e., six cross‐sectional data collections by 2022). To be included in QWL, respondents had to work full‐time or part‐time, or to be on temporary leave at the time of data collection (e.g., parental leave) [26]. Over this period, 9,246 respondents from 471 of 539 occupations (excluding military codes) based on Census codes provided data in QWL.

We selected the item “During the past 30 days, for about how many days did your poor physical or mental health keep you from doing your usual activities, such as self‐care, work, or recreation?” for our health outcome variable. Respondents chose a number between 0 and 30.

### 2.2 Criteria for Successful Matching and Statistical Analysis

Using *R*‐Studio, we checked three quality criteria in each step of the matching procedure detailed in the Overview section of the Introduction (see also Figure 1): The first criterion was the number of jobs that were matched by job code and job title. To evaluate this criterion, we inspected frequencies and percentages of matched jobs.

The second criterion was the availability and consistency of information for matched jobs. Regarding availability, we inspected the frequency and percentage of matched jobs for which none of the information of interest was available (i.e., missing values). The consistency of information was evaluated when more detailed coding systems were matched to less detailed ones; in these cases, we evaluated whether very heterogeneous information from the more detailed system would be summarized in less‐detailed codes. For that purpose, we calculated means, standard deviations, and ranges within the less‐detailed job codes. We also reported these measures at an aggregated level to understand to what extent jobs within a specific group varied from each other.

The third criterion was the extent to which available information varied between groups that we identified to be significant by criteria 1 and 2 (e.g., matched vs. unmatched jobs, jobs with vs. without full information). For that purpose, we compared these groups regarding relevant information using inferential statistics that aligned with the number of groups and the scale levels of the variables of interest. More specifically, we compared two groups (e.g., matched vs. unmatched job codes or workers in these groups) using Welch's *t*‐test to compare metric variables (e.g., the level of job control) and *χ* ^2^‐tests to assess distributions over nominal variables (e.g., having a college degree). When necessary, we created more than two groups (see Step 1) and compared them via analyses of variance along with post hoc tests based on Bonferroni‐corrected *p*‐values.

## 3 Matching Procedure and Resulting Datasets

### 3.1 Step 1: Creating Simplified Exposure Data

The goal of this step was to establish a linkage that can be used to match O*NET‐19 codes with the 2010 Census codes used in ACS and GSS (i.e., preparing O*NET data for matching). As there is no direct crosswalk from O*NET‐19 to Census codes, we had to perform and build such a crosswalk in three steps: We first performed a crosswalk from O*NET‐19 codes to O*NET‐10 codes (i.e., the internal crosswalk) [27]. Then we created SOC‐10 codes by removing the O*NET extension and finally merged these SOC‐10 codes to Census codes (i.e., the external crosswalk) [28], resulting in *simplified exposure data*. The number of job codes of these crosswalks can be found at the top of Table 1.

**Table 1:** Descriptive and inferential statistics on O*NET for crosswalks between O*NET‐19 codes and census codes (Step 1).

	O*NET‐19 codes	Census codes
Job codes in raw crosswalks	993^a^	535^b^
O*NET‐10 codes	1,091^a^	^—^
SOC‐10 codes	822	551^b^
Information on job control^c^		
Job control available	892	^—^
*M* (SD) O*NET job control	73.23 (10.54)	^—^
Direct matches	347	348
Title matched	320	320
Job control available	330	331
*M* (SD) O*NET job control	70.27 (10.57)	70.28 (10.55)
Split Census matches	217	72
Job control available	168	63
*M* (SD) O*NET job control	75.62 (8.23)	74.47 (8.29)
Unmatched job codes	445	115
Job control available	393	—
*M* (SD) O*NET job control	74.69 (10.87)	—
Comparison regarding job control^d^	*F*_(2, 889)_ = 22.07, *p* < 0.001	*t*(104) = −3.52, *p* < 0.001

*Note:* O*NET = Occupational Information Network. O*NET‐10 codes = 2010 Standard Occupational Classification codes with O*NET‐extension. O*NET‐19 codes = 2019 Standard Occupational Classification codes with O*NET‐extension. SOC‐10 codes = 2010 Standard Occupational Classification codes. *M* = mean. SD = standard deviation.

^a^ Included in the crosswalk from O*NET‐19 to O*NET‐10 codes.

^b^ Included in the crosswalk from SOC‐10 to Census codes.

^c^ Based on O*NET version 29.0 after the merge with the crosswalks. Note that this number exceeds the number of jobs with available data in the raw O*NET file (*n* = 879) because of O*NET‐19 matches that were matched and combined with multiple Census codes.

^d^ To compare direct matches, split Census matches, and unmatched job codes at the level of O*NET‐19 codes regarding job control, we conducted an analysis of variance; the post hoc comparison indicated that the job control scores of direct matches were significantly lower than both the split Census matches (*p* < 0.001) and unmatched O*NET‐19 codes (*p* < 0.001). To compare direct matches and split Census matches at the level of Census job codes regarding job control, we conducted Welch's *t*‐test.

To evaluate this three‐step crosswalk, we inspected how job codes distributed over three types of matches: direct matches (i.e., a single O*NET‐19 code was directly matched to one Census code)^6^; split Census matches (i.e., many O*NET‐19 codes were matched to a single Census code); and unmatched job codes (i.e., O*NET‐19 codes were not matched to any Census codes and Census codes were not matched to any O*NET‐19 codes; Criterion 1). After inspecting information on job control of direct matches and split Census matches more closely (Criterion 2), we compared O*NET job control between these three types (Criterion 3).

#### 3.1.1 Criterion 1: The Number of O*NET‐19 Codes That Can Be Cross‐Walked to Census Codes

As shown in Table 1, we identified direct O*NET‐19 matches for 348 (65.05%) Census codes (see Table 2 on pp. 13–22 in Online Supplement A).^7^ Inspecting the job titles of the direct matches, we found that 28 O*NET‐19 job titles were different from the matched Census job titles. These differences were based on minor differences in spelling or punctuation, minor changes in wording, extensions, or completely different job titles (see Table 1 on pp. 1–3 in Online Supplement A). For example, O*NET‐19 classified “Fast Food and Counter Workers” (35‐3023.00) while the Census described “Combined food preparation and serving workers, including fast food” (4050). It is clear to reviewers these two are a match, but if an automated system is used for creating a crosswalk, they may be deemed as unmatched. Based on further exploration, we found that in 23 direct matches, the job title was the same across O*NET‐10, SOC‐10, and Census but differed from O*NET‐19 (in only three cases, the job titles of O*NET‐10 and SOC‐10 aligned with O*NET‐19 but not with Census). Additionally, for two direct matches with different job titles, O*NET‐10 more strongly differentiated than O*NET‐19 or Census (e.g., First‐Line Supervisors of Firefighting and Prevention Workers from O*NET‐19 were matched with three distinct O*NET‐10 codes but only one SOC‐10 code and one Census code, respectively).

The split Census matches consisted of 217 (21.85%) O*NET‐19 codes and 72 (13.46%) Census codes (see also Table 3 on pp. 23–29 in Online Supplement A). We found that 445 (44.81%) O*NET‐19 codes (see pp. 4–11 in Online Supplement A) and 115 (21.50%) Census codes (see pp. 11–13 in Online Supplement A) were unmatched (Table 1).

#### 3.1.2 Criterion 2: Availability and Consistency of Information on O*NET Job Control in Matched O*NET‐19 and Census Codes

In total, information on O*NET job control was available for 394 Census codes (331 direct matches, 63 split matches). For split Census matches, we first inspected the information density (i.e., percentage of O*NET‐19 codes per Census code that provide information on O*NET job control). Within a single Census code, on average, 73% of O*NET‐19 codes provided information on job control. At least one O*NET job control estimate was available for 63 out of 72 Census codes (87.5%), and for the remaining nine Census codes, none of the 18 matched O*NET‐19 codes provided information on job control. For 39 of the 63 Census codes with job control information, all matched O*NET‐19 codes (*n* = 101) provided information on job control.

To understand how much O*NET‐19 job control estimates that were combined into a single Census code differed from each other, we inspected the range of O*NET job control within the Census code if at least two O*NET‐19 codes included that information. The minimum range of O*NET job control within a single Census code was 0.14 (for the Census code 3150 Occupational therapists), indicating almost the same level of job control for O*NET‐19 codes that matched this Census code. The maximum range was 26.96 (for the Census code 4460 Embalmers and funeral attendants). The average range of O*NET job control within Census codes was 8.49 (*SD* = 5.95).

#### 3.1.3 Criterion 3: Differences Between Direct Matched, Split Matched, and Unmatched Job Codes in O*NET Job Control

To compare direct matches, split Census matches, and unmatched O*NET‐19 codes in grading O*NET job control, we conducted an analysis of variance (i.e., comparison at O*NET‐19‐code level; see the O*NET‐19 column in Table 1). The comparison indicated a significant difference between the three groups, *F*_(2, 889)_ = 22.07, *p* < 0.001. As a post‐hoc test, we conducted a pairwise *t*‐test with Bonferroni‐corrected *p*‐values showing that the job control scores of direct matches were significantly lower than the scores of both the split Census matches (*p* < 0.001) and unmatched O*NET‐19 codes (*p* < 0.001).

Additionally, we compared the direct matches to the split Census matches for which we aggregated job control for each Census code (i.e., a comparison at Census‐code level; see the Census column in Table 1). Welch's *t*‐test indicated that the level of job control was significantly higher among split Census codes compared to direct matches, *t*(104) = −3.52, *p* < 0.001.

#### 3.1.4 Summary and Reflection for Step 1

For almost 80% of the Census codes, at least one matching O*NET‐19 code was available. However, not all of these matches may be as reliable as expected: Some job titles did not match, and some job control ranges among the split Census matches were quite large, which means that using averages of O*NET job control scores might introduce exposure misclassification and information bias that potentially affect subsequent analysis. Thus, we advise readers interested in matching O*NET‐19 to data that include SOC‐10 codes to prefer them over Census codes (since SOC‐10 codes are more detailed than Census and closely related to O*NET‐19) and skip the next steps.

Another problem was that almost half of O*NET‐19 codes could not be matched to a Census code. Thus, we cannot use them in further analyses (e.g., to draw conclusions on the sociodemographic composition of these occupations).

Finally, O*NET job control was significantly lower for direct matches compared to split Census matches and unmatched O*NET‐19 codes, suggesting that directly matched O*NET‐19 codes describe occupations that are less likely to be academic or involve leadership responsibility. The resulting simplified exposure data included 993 O*NET‐19 codes and 535 Census codes; most of these occupations had information on job control.^8^

### 3.2 Step 2: Creating Occupation Data

In this step, we matched simplified exposure data from Step 1 (i.e., the direct matches, split matches, and unmatched Census codes) with occupational sociodemographics from ACS (i.e., proportion of workers with certain sociodemographic characteristics in each job; e.g., the percent of women on the job), resulting in *occupation data*. As occupational sociodemographics, we selected age, income, sex × race/ethnicity, and education, as these characteristics are commonly reported in the occupational health literature [29, 30]. We further decided to investigate sex × race/ethnicity rather than isolated effects of each characteristic following intersectionality reasoning. Based on Crenshaw [31], previous research found combined effects of sex and race to be crucial for understanding occupational and health inequalities [32, 33, 34]. For clarity, we will refer to Census codes from Step 1 as O*NET‐Census codes and those from ACS as ACS‐Census codes.

We evaluated the number of matches (Criterion 1) and the extent to which information on job control and workforce sociodemographics was available for matched data (Criterion 2). Then we compared matched and unmatched Census codes on job control (Criterion 3).

#### 3.2.1 Criterion 1: Number of Matched Job Codes From O*NET and ACS

Out of 535 total O*NET‐Census codes from Step 1, 475 (88.8%)^9^ could be matched to ACS, which corresponded to 497 O*NET‐19 codes because of split matches. The rest, 60 O*NET‐Census codes (corresponding to 62 O*NET‐19 codes), could not be matched to ACS (see pp. 33–34 in Online Supplement A).

Among the 475 matched Census codes, we discovered 45 codes in which the wording of the job title varied between O*NET‐Census and ACS‐Census codes. These variations were due to dissimilar wording for “miscellaneous” occupations, minor spelling or punctuation differences, and content‐related differences (see Table 4 on pp. 30–32 in Online Supplement A).

#### 3.2.2 Criterion 2: Availability of Job Control and Sociodemographic Information in Matched and Unmatched Job Codes

Information on both job control (O*NET) and occupational sociodemographics (ACS) was available for 341 matched Census codes, and for the remaining matched Census codes (*n* = 134) only occupational sociodemographics was available. Of the 60 unmatched Census codes, 53 had job control data on O*NET, but no sociodemographic information could be linked.

#### 3.2.3 Criterion 3: Differences Between Matched and Unmatched Job Codes on Job Control and Occupational Sociodemographics

The comparison between matched and unmatched O*NET‐Census codes indicated no significant difference in job control (*t*(77.4) = −1.02, *p* = 0.312; see Table 2). As all ACS‐Census codes could be matched to at least one O*NET‐Census code, comparing matched and unmatched ACS‐Census codes on sociodemographics was not possible. Thus, we will describe them: As shown in Table 2, data from 8,704,286 workers were included in the 475 Census codes in ACS. On average, the mean age of these occupations was 42.92 years, and the mean annual income was $45,556. Within Census codes, non‐Hispanic white men had the highest average share (43.49%), followed by non‐Hispanic white women (27.82%), ethnic minority men (16.64%), and ethnic minority women (12.04%). The share of workers without a college degree in the workforce was 66.89% on average.

**Table 2:** Descriptive and inferential statistics on O*NET job control and workforce characteristics for matched and unmatched job codes (Step 2).

	Matched	Unmatched (O*NET)
*N*_Census codes_	475	60
*N*_O*NET‐19 codes_	497	62
O*NET job control		
Available	341	53
*M* (SD)	71.13 (10.55)	69.77 (8.77)
Range	39.55–92.17	51.66–89.76
Comparison	*t*(77.4) = −1.017, *p* = 0.312	
Workforce characteristics (ACS)		
*n*_people_	8,704,286	—
*M* (SD) average age	42.92 (4.36)	—
*M* (SD) maximum age within job	90.48 (5.53)	—
*M* (SD) average income	$45,556 (26,988)	—
*M* (SD) maximum income within job	$610,920 (165,858)	—
Sex × race/ethnicity: *n* (occupation mean %)		
Non‐Hispanic white male	3,201,121 (43.49%)	—
Non‐Hispanic white female	2,914,614 (27.82%)	—
Ethnic minority male	1,316,448 (16.64%)	—
Ethnic minority female	1,272,103 (12.04%)	—
Education: *n* (occupation mean %)		
No college degree	5,796,191 (66.89%)	—
At least a college degree	2,908,095 (33.11%)	—

*Note:* ACS = American Community Survey. O*NET = Occupational Information Network. *M* = mean. SD = standard deviation. *n*_Census codes_ = number of Census Occupation Codes. *N*_O*NET‐19 codes_ = number of 2019 Standard Occupational Classification codes with O*NET‐extension. *n*_people_ = number of people.

#### 3.2.4 Summary and Reflection for Step 2

Matching two datasets by Census codes, O*NET‐matched Census codes from Step 1 and ACS‐Census codes, the share of matched job codes in this step is higher than in the previous step. However, we were surprised to find differences in the job titles. Those who use text‐matching strategies should be aware of these differences. The most important finding in this step was that for most occupations in ACS (i.e., 341 Census codes), data on both O*NET job control *and* occupational sociodemographics were available. While these occupational data are based on more than eight million workers, we want to emphasize that several occupations from the O*NET crosswalk could not be matched in this or the previous step, leaving us without information on their sociodemographic composition. Readers who want to build an occupation‐level dataset (e.g., for studying how exposure is distributed over age groups), may skip the next step and treat the occupation as the unit of analysis.

### 3.3 Step 3: Enriching Worker Health Data

In this final step, we matched the occupation data (i.e., 475 Census codes for which we matched O*NET and ACS) from Step 2 to GSS. That means that every participant in GSS was assigned the respective occupational information through their Census code. The idea behind this third step is that we enrich individual workers’ health data by information on objective job control and occupational sociodemographics, resulting in the type of dataset ultimately necessary in order to investigate health effects of objective occupational exposure in a general population.

We evaluated the resulting dataset by the number of jobs and participants for whom (a) a matching Census code (Criterion 1), information on (b) O*NET job control, and (c) occupational sociodemographics were available (Criterion 2). Then we compared workers' health and sociodemographic data between Census codes where information on job control was available versus not available (Criterion 3).

#### 3.3.1 Criterion 1: Number of Workers for Whom Occupational Information Can Be Matched

The GSS data included 9,246 working adults nested in 471 Census codes. Forty‐five (< 1%) workers were nested in 25 Census codes that could not be matched to occupation data (see Figure 2 and p. 42 of Online Supplement A). Since the group of workers whose Census codes could not be matched was considerably smaller than the group of workers with matchable Census codes, we did not compare them using inferential statistics.

**Figure 2:**
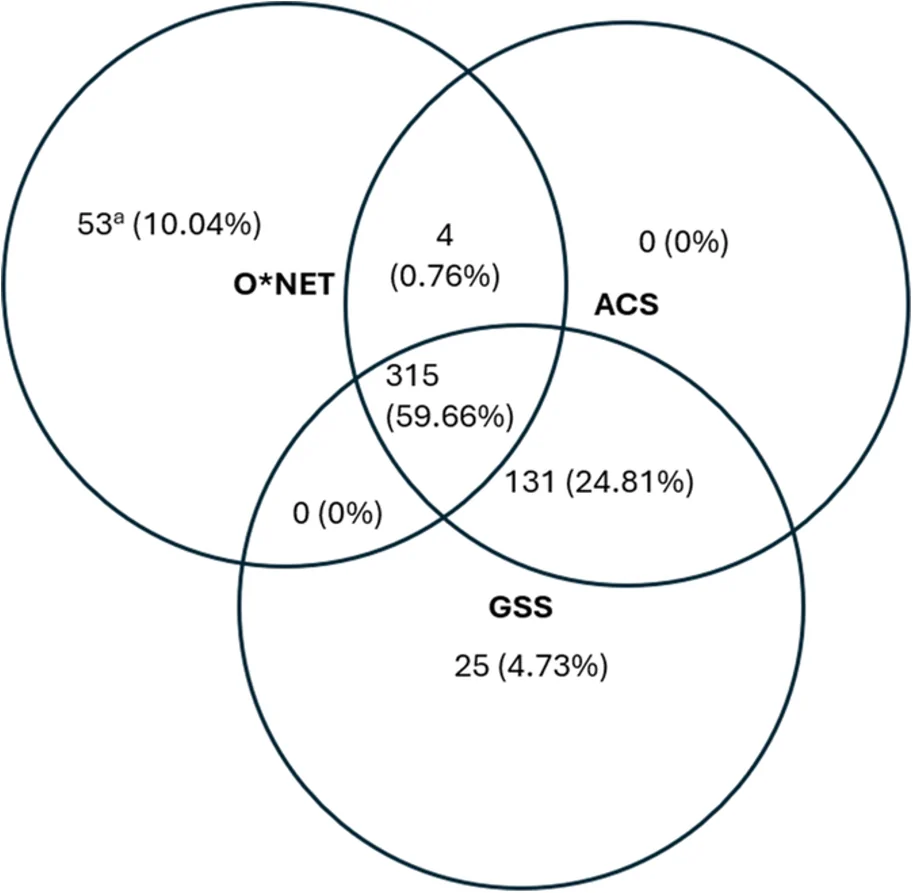
Venn diagram that represents the number of Census codes with available information in Step 3 (and Step 2; *n* = 528). *Note*: ^a^ This number refers to the number of Census codes with information on job control that could not be matched to ACS in Step 2. ACS, American Community Survey; GSS, General Social Survey; O*NET, Occupational Information Network.

#### 3.3.2 Criterion 2: Availability of Job Control and Occupational Sociodemographics

Among the 9,201 matched workers, O*NET job control was available for 5,967 workers in 315 Census codes (see pp. 45–50 of Online Supplement A). Thus, for the remaining 3,279 (35.46%) workers in 131 Census codes, only information on workforce sociodemographics but not on O*NET job control was available (see pp. 42–45 of Online Supplement A), resulting in 446 Census codes with information on workforce sociodemographics.

#### 3.3.3 Criterion 3: Differences Between Census Codes With and Without Job Control Data Regarding Occupational Sociodemographics and Self‐Reported Data

To investigate this criterion, we first compared *occupational* sociodemographic data from ACS between Census codes with available versus unavailable occupational exposure data. As shown in Table 3, we found no significant differences in the mean age and both mean and maximum income. We found that the maximum age was significantly higher in Census codes without occupational exposure data. Chi‐square tests also indicated significant differences in the availability of occupational exposure data comparing both sex × race/ethnicity groups and education groups. More specifically, more non‐Hispanic white men and fewer non‐Hispanic white women than expected worked in Census coded‐work for which occupational exposure data were available; college graduates were more likely to work in a Census‐code job without available occupational exposure data.

**Table 3:** Descriptive and inferential statistics on workforce demographics comparing Census codes with and without complete occupational exposure data (Step 3).

	Complete data on occupational exposure		Comparison
	Available	Not available	
*n*_people ACS_	5,667,535	2,998,829	*χ* ^2^(1, *n* = 8,666,364) = 821,797, *p* < 0.001
*n*_*Census codes*_	315	156^a^	*χ* ^2^(1, *n* = 471) = 53.68, *p* < 0.001
*M* (SD) average age	42.9 (4.52)	42.9 (4.10)	*t*(267) = −0.001, *p* = 0.999
*M* (SD) maximum age^b^	90.28 (5.63)	92.16 (3.88)	*t*(347) = 4.05, *p* < 0.001
*M* (SD) average income	$45,617.39 (26,917.37)	$44,027.42 (26,417.9)	*t*(247) = −0.58, *p* = 0.565
*M* (SD) maximum income^b^	$614,450.8 (165,469.2)	$640,435.1 (137,494.1)	*t*(290) = 1.71, *p* = 0.089
Sex × race/ethnicity: *n* (occupation mean %)			
Non‐Hispanic white male	2,169,548 (42.57%)	1,011,369 (43.93%)	*χ* ^2^(3, *n* = 8,666,364) = 20,189, *p* < 0.001
Non‐Hispanic white female	1,827,708 (28.74%)	1,079,351 (27.04%)	
Ethnic minority male	856,662 (16.26%)	452,890 (17.24%)	
Ethnic minority female	813,617 (12.43%)	455,219 (11.79%)	
Education: *n* (occupation mean %)			
No college degree	3,896,719 (68.14%)	1,874,642 (64.13%)	*χ* ^2^(1, *n* = 8,666,364) = 34,355, *p* < 0.001
At least a college degree	1,770,816 (31.86%)	1,124,187 (35.87%)	

*Note: M* = mean. SD = standard deviation. *n*_people ACS_ = number of people in the American Community Survey. *n*_Census codes_ = number of Census occupation codes.

^a^ These 156 Census codes include 131 Census codes, for which only information on workforce demographics is available, and 25 Census codes, for which neither information on occupation‐level job control nor workforce sociodemographics is available.

^b^ Within a Census code.

We inspected individual‐level *self‐report* data from GSS and compared them between workers with and without available occupational exposure data. As shown in Table 4, days of poor health and levels of self‐reported income did not vary between workers with and workers without occupational exposure data. Yet, workers for whom information was available were significantly younger. More non‐Hispanic white men and less non‐Hispanic white women than expected worked in Census codes with occupational exposure data, which resembles our findings on sociodemographics at the occupation level. Similarly, workers without a college degree were more likely to work in Census codes with available occupational exposure data.

**Table 4:** Descriptive and inferential statistics on self‐reported information comparing workers with and without complete occupational exposure data (Step 3).

	Complete data on occupational exposure		Comparison
	Available	Not available	
*n*_*people GSS*_	5967	3279	*χ* ^2^(1, *n* = 9,246) = 781.46, *p* < 0.001
*M* (SD) days of poor health	1.65 (4.74)	1.53 (4.62)	*t*(6,776) = −1.23, *p* = 0.219
*M* (SD) age	42.69 (13.59)	43.31 (13.69)	*t*(6,619) = 2.08, *p* = 0.038
Income: *n* (%)^a^			
< $25,000	1021 (12.02%)	554 (6.52%)	*χ* ^2^(1, *n* = 8,494) = 0.05, *p* = 0.824
≥ $25,000	4462 (52.53%)	2457 (28.93%)	
Sex × race/ethnicity: *n* (%)^a^			
Non‐Hispanic white male	2123 (23.04%)	988 (10.72%)	*χ* ^2^(3, *n* = 9,216) = 34.40, *p* < 0.001
Non‐Hispanic white female	1941 (21.06%)	1208 (13.11%)	
Ethnic minority male	868 (9.42%)	460 (4.99%)	
Ethnic minority female	1013 (10.99%)	615 (6.67%)	
Education: *n* (%)^a^			
No college degree	4115 (44.51%)	1998 (21.61%)	*χ* ^2^(1, *n* = 9,245) = 60.72, *p* < 0.001
At least a college degree	1851 (20.02%)	1281 (13.86%)	

*Note: M* = mean. SD = standard deviation. *n_people GSS_* = number of GSS participants.

^a^ Percentages are calculated based on the total number of GSS participants with valid responses on the respective questions.

#### 3.3.4 Summary and Reflection for Step 3

For most workers, matching job control and occupational sociodemographic information to their Census codes was possible. However, complete occupation data was available for only 65% of the workers. Although each crosswalk and dataset included all Census codes and allowed matching of them, an analysis that considers the complex interplay of both job control and occupational sociodemographics may return biased results because data availability and accuracy vary by the worker's personal characteristics.

The resulting dataset differed from the datasets of the previous steps with respect to the level of analysis: While the datasets from Steps 1 and 2 were purely focused on occupations as units of analysis, this dataset can be classified as multi‐level data with individual workers nested in occupations.

## 4 Discussion

Matching multiple datasets allows researchers to combine a large variety of information not otherwise available in a single dataset. However, our matching shows that this variety might come at the costs of omitting some jobs and workers: We started with 993 O*NET‐19 codes in Step 1 and ended with 315 Census codes in Step 3. Although several O*NET‐19 codes were summarized by a single Census code, we cannot claim that our final dataset fully represents the original 993 O*NET‐19 codes. In an ideal world, matching multiple datasets would result in a larger set of information without reducing the number of jobs and workers.

However, we found that enriching datasets involves several issues related to the nature of crosswalks and the variables of interest.^10^ Regarding the nature of crosswalks, we lost 429 O*NET‐19 codes and can say nothing about the sociodemographic composition or health status of workers in these jobs. The explanations for the matching problems we encountered stem from the different purposes for which each dataset was created. While O*NET provides guidance to job seekers who might be interested in the variety of jobs reflected by the detailed O*NET‐SOC coding system [35], ACS is supposed to describe and summarize populations engaging in economic activity categorized at a less‐detailed level [36], reflecting differences in what is considered a meaningful definition of an “occupation” for each purpose. Thus, matching both datasets requires crosswalks.

Regarding the variables of interest (in our case, job control), two issues may arise. First, when combining job codes of a more detailed coding system (here, O*NET‐19 codes) with a less detailed one (here, Census codes), values on that resultant variable might vary considerably. Second, the datasets may not provide complete information, as in the case of O*NET job control that was not available for a large number of GSS participants (some due to missing data in O*NET and matching problems).

Under these circumstances, it is not possible to determine whether the datasets or crosswalks are biased in favor of one or another sociodemographic group. What we can say, however, is that direct matches between O*NET‐19 codes and Census codes were characterized by significantly lower levels of job control, and that non‐Hispanic white men were more likely to work in an occupation with available information on job control. Our analysis suggests that if we restricted the final dataset to contain only the people who had direct‐match jobs (i.e., O*NET‐19 codes that match single Census codes), then any investigation on job control using the dataset would inevitably over‐represent non‐Hispanic white men and workers without a college degree. Those who engage in data merging should keep in mind that this finding is specific to our exposure of interest, job control. If other occupational exposure data were to be imputed from O*NET, the under‐ or over‐representation in the final sample may be different.

Since missing data might result from the matching process, it is crucial to think about potential solutions. Researchers should first inspect and describe the extent to which data are missing and the reason why they are missing—because job codes need to be dropped or because the original data did not provide any data on that code. The data that is available may help determine whether the probability of data being missing is random or not (e.g., using the mice package in *R* [37, 38]. Depending on the results and the role of the occupation data, researchers might then consider various options for dealing with missingness, such as omission, multiple imputation [39] or inverse‐probability‐weighted estimation [40] (for guidance on these options, see Perkins et al. [41]).

As previously mentioned, the individual workers in the final dataset are clustered by Census codes [14]. That means that occupational exposure and workforce sociodemographics are the same for all workers who share the same Census code. Consequently, this structure should be accounted for in the analyses (e.g., by deriving cluster‐robust standard errors or applying multi‐level models) and can be used to investigate relationships between these occupation‐level variables and individual‐level variables [42]. For example, the data resulting from these three steps can be used to investigate whether the workforce composition (e.g., high vs. low shares of non‐Hispanic white men in the workforce) modifies gender differences in terms of health.

### 4.1 Implications for Research—A Checklist

Despite the problems raised by our findings, we do not expect a viable alternative to become available in near future. Instead of discouraging researchers from combining multiple datasets, we provide a checklist that researchers might apply when merging datasets. This checklist is not restricted to the datasets and variables we merged in this commentary and might support review processes (see Figure 3).

**Figure 3:**
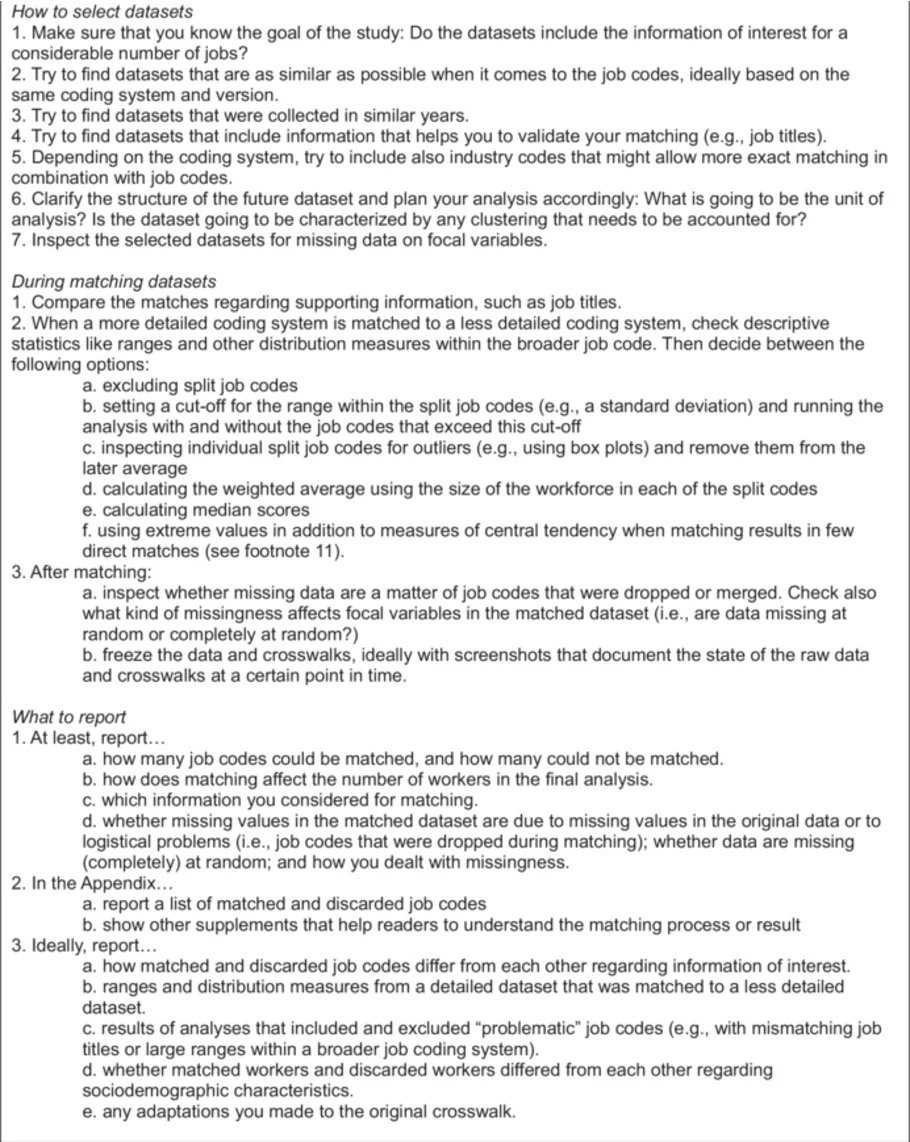
A checklist for researchers and reviewers.

The checklist considers three stages of the matching process—*selecting*, *matching*, and *reporting*. The first stage—*selecting* datasets—should be guided by the research interest. Ideally, datasets should align with one another in the coding system and version, as both might introduce logistic problems. If coding systems do not align, researchers should ensure that datasets include information that allows them to validate matches (e.g., job titles), and that they are ideally from the same time period, reflecting a similar occupational structure.

This information becomes particularly important at the second stage—the actual *matching*. Researchers might, for example, harmonize job titles from both datasets in capitalization, compare them to each other automatically, and then inspect job codes with mismatching job titles more closely. Especially when researchers want to match a detailed coding system to a less detailed one, they should check distribution measures (e.g., standard deviations, ranges) of the variable of interest. Simply using the average of this variable might affect subsequent analysis by introducing exposure misclassification and corresponding information bias. Thus, researchers must decide whether they want to: (a) include split job codes; (b) set a cut‐off for the range within the split job codes; (c) inspect individual split job codes for outliers they can remove from the later average; (d) calculate the weighted average using the size of the workforce in each of the split codes (see Fujishiro and Koessler [43]); (e) calculate median scores instead of mean scores to reduce the influence of extreme values; or (f) use extreme values (e.g., the 90th percentile) in addition to measures of central tendency.^11^

We further recommend freezing the datasets and crosswalks used [44]. This suggestion is based on our experience with ACS where the coding system was adapted to fit other Census codes in Summer 2025 without notifying the users of the online tool or renaming the variable (see Online Supplement C). While we appreciated this adaptation as it reduced mismatches in Step 2, we did not expect the change and would have preferred a choice between both the old and the new version.

Finally, at the *reporting* stage, researchers should make sure they indicate how many jobs were matched, and what information was used for matching without being too exhaustive. Much of this information (e.g., a list of job codes with different job titles) might be reported in an appendix.

### 4.2 Limitations and Future Research

Our results using the O*NET apply to research on job control. However, O*NET includes data on various other occupational exposures [14, 45], such as emotional [46] and physical labor [47], and characteristics of the physical work environment [48]. These data might be useful in the context of other research questions. Researchers who are interested in using occupational exposures from O*NET other than job control should re‐evaluate the quality of resulting matches with respect to the availability of data and variation between matched and unmatched jobs using our checklist.

Another limitation is that we matched our datasets using Census codes as the central coding system because of their availability in the health data. This limitation is related to Census codes being included in two out of three datasets we used and to the crosswalk in which only a share of the SOC and Census codes were direct matches. The explanation for this phenomenon is that Census codes are less detailed than SOC codes, which aligns with the purpose of describing jobs [5]. Thus, future research on other datasets should explore matching based on other coding systems, such as SOC. Alternatively, researchers might revise the crosswalk or experiment with more complex combinations of coding systems, such as Census occupation codes with Census Industry codes or North American Industrial Classification System (NAICS) codes [49, 50]. This suggestion is based on our impression that the more detailed job titles also included information on specific industries. For example, we matched the Census code for cooks (4020) to O*NET‐19 codes for cooks, fast food (35‐2011), institution and cafeteria (35‐2012), private household (35‐2013), restaurant (35‐2014), short order (35‐2015), and all other (35‐2019) [9]. If the research question concerns, for example, food safety practices, considering nuances between job settings by including an industry coding system might be worthwhile. The NAICS distinguishes, for example, fast‐food restaurants (722513), cafeterias (722514), private household (814110), and full‐service restaurants (722511) [49]. Thus, aside from the chosen variables, our choice of datasets and crosswalks limits the generalizability of our findings. To study this generalizability, researchers might follow and potentially extend the procedure outlined in our checklist.

Combining these avenues for future research suggests a need for meta‐research on job codes and occupational databases. More specifically, occupational health scientists from various disciplines might synthesize studies that match O*NET data on various exposures (e.g., in methodological reviews) and collect matching problems associated with these specific exposures. Across multiple exposures, general problem patterns might become visible and could be considered when constructing new crosswalks (e.g., by matching certain codes no longer to each other or adding warning messages concerning specific matches or matching regarding specific exposures). This meta‐research should engage in a dialogue with the institutions who provide coding systems and occupational data. These institutions might on their side integrate this knowledge as well as updates to the coding in their documentation. Since researchers all over the world use these systems and potentially draw important conclusions for policy makers from them, this approach might be extremely fruitful.

## 5 Conclusion

As collecting objective data on occupational exposures for each study would be expensive and time‐consuming, merging datasets provides an economically viable option for a wider scope of occupational health research. However, our analysis shows that this seemingly straightforward solution has several pitfalls that might affect the generalizability and accuracy of research findings. Therefore, we made several recommendations that might support researchers and reviewers in estimating how much merging datasets affects their findings.

## Author Contributions

**Franziska J. Kößler:** conceptualization, methodology, software, formal analysis, data curation, writing – original draft, writing – review and editing, visualization. **Candice Y. Johnson:** methodology, writing – reviewing and editing. **Amel Omari:** writing – reviewing and editing. **Kaori Fujishiro:** conceptualization, methodology, writing‐review and editing, supervision.

## Funding

The authors have nothing to report.

## Ethics Statement

The authors have nothing to report. Data used in this manuscript are all within the public domain.

## Consent

The authors have nothing to report.

## Conflicts of Interest

The authors declare no conflicts of interest.

## Data Availability

The data that support the findings of this study are available in multiple sources at https://www.onetcenter.org. These data were derived from the following resources available in the public domain: Occupational Information Network, https://www.onetcenter.org; IPUMS USA, https://sda.usa.ipums.org/sdaweb/analysis/?dataset=all_acs_samples; General Social Survey, http://gss.norc.org.

## Footnotes

1 For readability purposes, we refer to them as SOC and Census codes in the following. Since we refer to different versions of SOC codes, we also add the respective year when necessary (e.g., SOC‐10 codes refer to 2010 SOC codes).

2 To enhance readability, we refer to 2019 O*NET‐SOC codes as ‘O*NET‐19 codes’ and 2010 O*NET‐SOC codes as ‘O*NET‐10 codes’ in the following.

3 As these three items stem from different domains the concrete meaning of *low* (1) and *high* (5) values varies between them. For item 4.A.2.b.3 IM, the scale ranges from *not important* (1) to *extremely important* (5); for both 4.C.3.b.8 and 4.C.3.a.4, scale ranges from *no freedom* (1) to *a lot of freedom* (5).

4 Note that we extracted this information two times—first in March 2025 and then in January 2026. We will report our findings based on the second extraction but will also mention differences to the first extraction in the discussion.

5 We excluded military occupations because O*NET does not provide information on them.

6 We categorized matches where multiple Census codes were matched with a single O*NET‐19 code as direct matches because these allow a clear information transfer. We found only one of these cases in our data, namely the O*NET‐19 code (45‐3031.00 Fishing and Hunting Workers) that matched two Census codes (6100 Fishers and related fishing workers, 6110 Hunters and trappers).

7 Please note that we moved our Online Supplement to PsychArchives: https://doi.org/10.23668/psycharchives.22519.

8 We decided to keep also unmatched codes to explore their further use.

9 Of the 475 matched Census codes in this step, 295 (62.11%) were direct matches in step 1 (see pp. 34–39 in Online Supplement A); 68 (14.32%) were split matches (see Table 5 on pp. 39–40 in Online Supplement A); and 112 (23.58%) were O*NET‐Census codes that could not be matched to O*NET‐19 codes in step 1 (see pp. 40–42 in Online Supplement A).

10 Note that similar problems may occur when combining different versions of same dataset.

11 Note that option f might be useful when the matching produces mostly split codes since the 90th percentile does not exist in direct matches.
